# Big Data in Biology and Medicine: Based on material from a joint workshop with representatives of the international Data-Enabled Life Science Alliance, July 4, 2013, Moscow, Russia

**Published:** 2013

**Authors:** O. P. Trifonova, V. A. Il’in, E. V. Kolker, A. V. Lisitsa

**Affiliations:** Orekhovich Research Institute of Biomedical Chemistry, Russian Academy of Medical Sciences, Pogodinskaya Str. 10, Bld. 8, Moscow, Russia, 119121; Scientific Research Center “Kurchatov Institute,” Academician Kurchatov Sq. 1, Moscow, Russia 123182; Skobel’tsyn Research Institute of Nuclear Physics, Lomonosov Moscow State University, Leninskie Gory 1, Bld. 58, Moscow, Russia, 119992; DELSA Global, USA; Seattle Children’s Research Institute, 1900 9th Ave Seattle, WA 98101, USA

## FORUM

The task of extracting new knowledge from large data sets is designated by the term “Big Data.” To put it simply, the Big Data phenomenon is when the results of your experiments cannot be imported into an Excel file. Estimated, the volume of Twitter chats throughout a year is several orders of magnitude larger than the volume of a person’s memory accumulated during his/her entire life. As compared to Twitter, all the data on human genomes constitute a negligibly small amount [1]. The problem of converting data sets into knowledge brought up by the U.S. National Institutes of Health in 2013 is the primary area of interest of the Data-Enabled Life Science Alliance (DELSA, www.delsaglobal.org) [2].

Why have the issues of computer- aided collection of Big Data created incentives for the formation of the DELSA community, which includes over 80 world-leading researchers focused on the areas of medicine, health care, and applied information science? This new trend was discussed by the participants of the workshop “Convergent Technologies: Big Data in Biology and Medicine.”

The total number of workshop participants was 35, including representatives of research institutes dealing with the analysis of large experimental data sets and commercial companies developing information systems. The workshop participants delivered 16 short reports that were aimed at discussing how manipulating large data sets is related to the issues of medicine and health care.

The workshop was opened by Prof. Eugene Kolker, who presented a report on the behalf of the Data-Enabled Life Science Alliance (DELSA, www.delsaglobal.org). The alliance supports the globalization of bioinformatics approaches in life sciences and the establishment of scientific communities in the field of “omics.” The main idea is to accelerate translation of the results of biomedical research to satisfy the needs of the community.

Large data sets that need to be stored, processed, and analyzed are accumulated in many scientific fields, in addition to biology; there is nothing surprising about this fact. Large data sets in the field of highenergy physics imply several dozen petabytes; in biology, this number is lower by an order of magnitude, although it also approaches petabyte scale. The question discussed during the workshop was what Russian researchers should focus on in the Big Data world: either molecular biology in the “omics” format, or integrative biology in brain modeling, or social sciences?

The tasks of working with large data sets can be subdivided into two groups: (1) when data are obtained interactively and need to be processed immediately and (2) when there is a large body of accumulated data requiring comprehensive interpretation. The former category of data is related to commercial systems, such as Google, Twitter, and Facebook. Repositories of genomic and proteomic data exemplify the latter type of data.

Systems for handling large data arrays are being developed at the Institute for System Programming, Russian Academy of Sciences, with special attention on poorly structured and ambiguous data that are typical of the medical and biological fields. Collections of software utilities and packages, as well as distributed programming frameworks running on clusters consisting of several hundreds and thousands of nodes, are employed to implement smart methods for data search, storage, and analysis. Such projects as Hadoop (http://hadoop.apache.org/), Data-Intensive Computing, and NoSQL are used to run searches and context mechanisms when handling data sets on a number of modern web sites.

**Fig:**
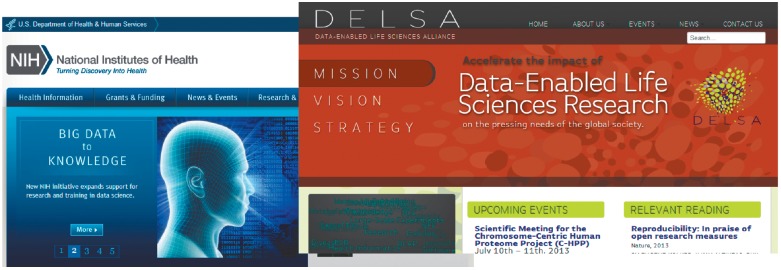
From problem to solution: the experts in the field of data processing, Data-Enabled Life Science Alliance-DELSA, are ready to beat back challenge of NIH

Prof. Konstantin Anokhin (Scientific Research Center “Kurchatov Institute”), talked on the fundamentally novel discipline of connectomics, which is focused on handling data sets by integrating data obtained at various organizational levels. Large bodies of data will accumulate in the field of neuroscience because of the merging of two fundamental factors. First, an enormous amount of results obtained using high-resolution analytical methods has been accumulated in the field of neurosciences. Second, the main concern of scientists is whole-brain functioning and how its function is projected onto the system (mind, thought, action), rather than the function of individual synapses. Obtaining data on the functioning of the brain as a system includes visualization techniques: high-resolution computed tomography, light microscopy, and electron microscopy. Megaprojects on brain simulation have already been launched (e.g., the Human Brain Project in Europe); the investments to obtaining new experimental data will be devalued with time, while the analysis of the resulting data will become the highest priority.

Extraction and interpretation of information from existing databases using novel analytical algorithms will play a key role in science in future. The existence of a large number of open information sources, including various databases and search systems, often impedes the search for the desired data. According to Andrey Lisitsa (Research Institute of Biomedical Chemistry, Russian Academy of Medical Sciences), existing interactomics databases coincide to no more than 55% [3]. The goal in handling large data sets is to obtain a noncontradictory picture when integrating data taken from different sources.

The concept of dynamic profiling of a person’s health or the state of an underlying chronic disease using entire sets of high throughput data without reducing the dataset to the size of the diagnostic biomarker panels is being developed at the Research Center of Medical Genetics of the Russian Academy of Medical Sciences. The description of a normal human tissue requires one to integrate several thousand quantifiable variables that may be derived using genome, transcriptome and/or proteome profiling techniques; composite, integrative measures may be used to quantify the distance that separate any two samples. However, as each human organism has both individual genetic predispositions and a history of environmentalal exposure, the traditional concept of averaged norm would not be appropriate for personalized medicine applications in its true sense. Instead, Prof. Ancha Baranova introduced the concept of a multidimensional space occupied by set of normal sample tissue and the tissue-specific centers within this space (“the ideal state of the tissue”). The diseased tissues will be located at a greater distance from the center as compared to healthy ones. The proposed approach allows one to abandon binary (yes/no) predictions and to show the departure of a given tissue sample as a point in an easily understandable line graph that places each sample in the context of other samples collected from patients with the same condition and associated with survival and other post-hoc measures.

Prof. Vsevolod Makeev (Institute of General Genetics, Russian Academy of Sciences) asserted in his report that we will be dealing with large data sets more frequently in the near future. There will be two types of data: data pertaining to the individual genome (the 1000 Genomes Project), which are obtained once and subsequently stored in databases to be downloaded when required. The second type of data pertains to the transcriptome or proteome analysis, which is conducted on a regular basis in order to obtain an integrative personal omics profile [4]. There are several providers of such data in the case of genomes; Russian laboratories can use these repositories and employ their own bioinformatics approaches to arrive at new results [5].

The flow of dynamic data for individuals (results of monitoring the parameters of the organism) will increase as modern analytical methods are adopted. Researchers will face the need for rapid processing of continuously obtained data and for transferring the information to repositories for further annotation and automated decision-making. There emerges the need for modifying the technology of data storage and transfer to ensure a more rapid exchange of information. Cloud services for storing and transferring large sets of data exist already (e.g., AmazonS3).

The development of more rapid methods of mathematical analysis also plays a significant role. The report delivered by Ivan Oseledets (Institute of Computational Mathematics, Russian Academy of Sciences) focused on the mathematical apparatus for compact presentation of multidimensional arrays based on tensor trains (tensor train format, TT-format). Multidimensional tasks constantly emerge in biomedical applications; the TT-format allows one to identify the key variables that are sufficient to describe the system or process under study.

Medical data need to be processed interactively so that a preliminary diagnosis can be made no later than several minutes after the data have been obtained. The “Progress” company is currently developing a system for remote monitoring of medical indicators using mobile devices and the cellular network for data transfer (Telehealth, report by Oleg Gashnikov). This method allows one to provide 24-hour out-of-hospital monitoring of a patient, which is supposed to reduce medical services costs in future. At this stage, techniques for forming alarm patterns are to be developed based on accumulated data; algorithms are to be modified for each patient.

The report on the problem of collecting and processing the geo location data that are accumulated by mobile network operators and collected by aggregators, such as Google, Facebook, and AlterGeo, appeared to lie beyond the workshop’s topic on the face of it. The lecturer, Artem Wolftrub (leading developer at Gramant Ltd.), reported that a number of papers have been published by a group led by Alex Pentland and David Laser (Massachusetts Institute of Technology) since 2009, where it has been substantiated that the analysis of geo data can be no less informative for predicting socially important diseases than the genome is. Environmental factors (the socalled exposome) play a significant role in the pathogenesis of multigene diseases. Data regarding the exposome can be obtained with a sufficient degree of detail by analyzing the relocations of a person, by comparing the general regularities of population migrations, and by identifying the patterns that correlate with health risks (e.g., development of cardiovascular diseases or obesity [6]).

In their discussions, the workshop participants mentioned the Watson supercomputer in various contexts. This supercomputer was designed by IBM to provide answers to questions (theoretically any questions!) formulated using the natural language. It is one of the first examples of expert systems utilizing the Big Data principle. In 2011, it was announced that the supercomputer will be used to process poorly structured data sets in order to solve medical and health care problems [7].

When analyzing the problem of Big Data in biology and medicine, one should note that the disciplines have been characterized by the accumulation of large data sets that describe the results of observations since the natural philosophy era. During the genomic era, the aim of data accumulation seemed to be understandable. However, as the technical aspect was solved and the genome deciphered, it turned out that the data was poorly related to the problems of health maintenance [8].

In the post-genomic era, biomedical science has returned back to the level of phenomenological description oriented towards data collection only, without an understanding of the prospect of its further interpretation. The Human Proteome Project is such an example: data for each protein are collected; however, it is not always a given that these data can be used in the applied problems of in-vitro diagnostics. Another example is the Human Connectome Project, which is aimed at accumulating data on signal transduction between neurons in expectation of the fact that having been accumulated to a certain critical level, these data will allow one to simulate human brain activity using a computer.

In summary, the workshop participants noted that the Big Data phenomenon is related to the newly available opportunity of modern technogenic media to generate and store data; however, there is no clear understanding as to the reason and purpose for the accumulation of such data. Russian scientists should primarily focus on analyzing Big Data so that the data array can be converted into hypotheses applicable for verification using a point-wise biochemical experiment. The task of getting acquainted with the data accumulated within the “Connectome” Project is bound to be the main direction of development at the Russian subgroup of DELSA.
